# ALCAPA syndrome, a rare cause of sudden cardiac death

**DOI:** 10.1007/s12471-023-01829-5

**Published:** 2023-11-28

**Authors:** Muniebur Rehman, Thijs Braber, Mohamed Mouden, Siert Knollema, Ahmet Güçlü

**Affiliations:** 1 Department of Cardiology, Isala Heart Centre, Zwolle, The Netherlands; 2 Department of Nuclear Medicine, Isala Heart Centre, Zwolle, The Netherlands

Anomalous left coronary artery from the pulmonary artery (ALCAPA) syndrome is associated with a high mortality rate if untreated before the 1st year of life [1, 2]. Cardiac imaging remains of crucial importance in diagnosing and eventually treating this rare anomaly [1–5].

**Fig. 1:**
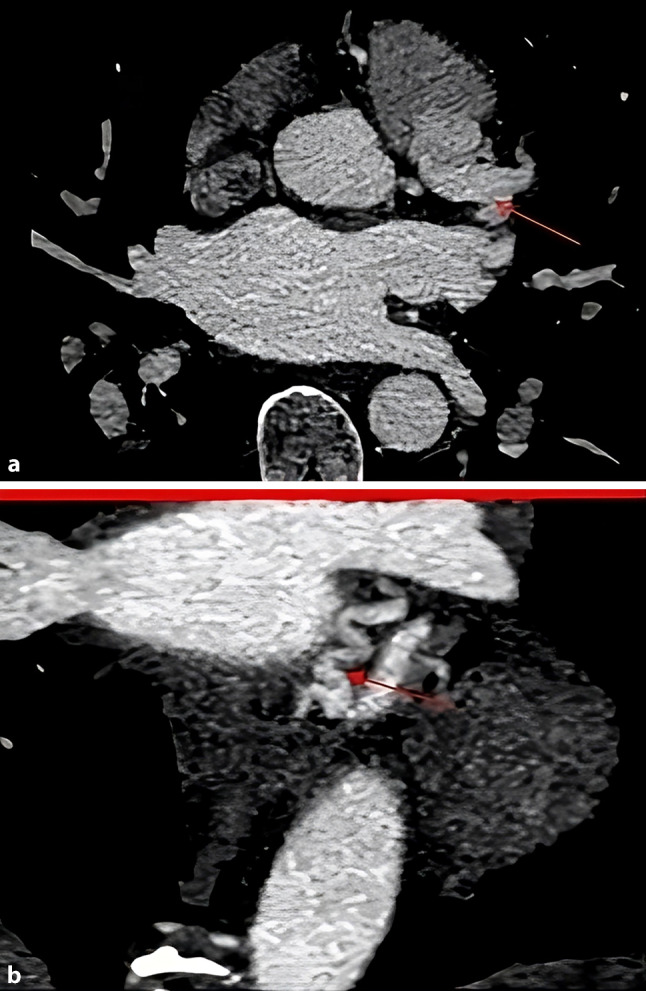
**a** Computed tomography angiography showed an anomalous origin of the left coronary artery (*LCA*, *red arrow*) from the pulmonary artery (*PA*), **b** an ectatic and tortuous LCA (*red arrow*)

A previously healthy 41-year-old woman was presented to our emergency ward after an out-of-hospital cardiac arrest. Return of spontaneous circulation was achieved after defibrillation at 200 J. The electrocardiogram showed no signs of cardiac ischaemia. However, due to the initial rhythm being ventricular fibrillation and wall motion abnormalities on the emergency transthoracic echocardiogram, emergency coronary angiography was performed, revealing an aneurysmal right coronary artery and collaterals extending to the left coronary artery. Due to these anatomical peculiarities, coronary computed tomography angiography was performed, which showed the presence of ALCAPA (Fig. 1a, b). The patient was referred to a surgical centre, where she underwent treatment.
